# Upadacitinib for Refractory Anti-melanoma Differentiation-Associated Gene 5 Antibody-Positive Dermatomyositis-Related Interstitial Lung Disease

**DOI:** 10.7759/cureus.105756

**Published:** 2026-03-24

**Authors:** Kohei Morikawa, Takao Nagashima, Hiroki Yabe, Hiroshi Kanazawa

**Affiliations:** 1 Division of Rheumatology, Department of Medicine, Jichi Medical University Saitama Medical Center, Saitama, JPN; 2 Division of Rheumatology and Clinical Immunology, Department of Medicine, Jichi Medical University, Shimotsuke, JPN; 3 Department of Rheumatology, Aomori Prefectural Central Hospital, Aomori, JPN

**Keywords:** calcineurin inhibitors, cyclophosphamide, interferon, interstitial lung disease, janus kinase inhibitor

## Abstract

We report a 54-year-old man with recurrent anti-melanoma differentiation-associated gene 5 (MDA5) antibody-positive dermatomyositis (DM)-associated interstitial lung disease (ILD) who was refractory to intensive triple therapy with high-dose glucocorticoids, tacrolimus, and intravenous cyclophosphamide. After relapse following treatment discontinuation, he developed worsening respiratory symptoms, elevated ferritin and Krebs von den Lungen-6 (KL-6) levels, and progressive ILD on computed tomography. Despite the reintroduction of triple therapy, his condition deteriorated. Upadacitinib was initiated at 15 mg/day and later escalated to 30 mg/day. Subsequently, respiratory symptoms improved, oxygen therapy was discontinued, inflammatory biomarkers declined, and radiologic findings markedly improved. Infectious complications, including bacteremia, cellulitis, and cytomegalovirus reactivation, were successfully managed with close monitoring. This case suggests that upadacitinib may be an effective add-on therapy for refractory anti-MDA5 antibody-positive DM-associated ILD when conventional intensive treatment is insufficient.

## Introduction

Anti-melanoma differentiation-associated gene 5 (MDA5) antibody-positive dermatomyositis (DM) is characterized by distinctive cutaneous features and a frequent complication of rapidly progressive interstitial lung disease (RP-ILD), often resulting in death [1]. Skin ulcers, particularly over Gottron’s sign, and keratotic palmar erythema, known as inverse Gottron’s sign, are characteristic cutaneous manifestations; mild muscle weakness, low creatine kinase (CK), and elevated serum ferritin are typical laboratory findings. Previous studies have reported mortality rates of approximately 30%-50% in patients with anti-MDA5 antibody-positive DM (MDA5-DM) complicated by RP-ILD, highlighting the need for effective rescue therapies. Intensive treatment strategies for MDA5-DM include glucocorticoids combined with two additional agents, such as rituximab, cyclophosphamide, intravenous immunoglobulin, mycophenolate, calcineurin inhibitors, and Janus kinase (JAK) inhibitors [2]. Nonetheless, treatment recommendations for MDA5-DM remain poorly established, and even early intensive triple-combination therapy with high-dose glucocorticoids, calcineurin inhibitors, and intravenous cyclophosphamide (IVCY) has produced mixed results [3,4].

The JAK family comprises JAK1, JAK2, JAK3, and tyrosine kinase 2 (TYK2), with each cytokine receptor consisting of two JAK isoforms. Recently, JAK inhibitors have emerged as a promising treatment option for patients resistant to intensive combination therapy [5,6]. JAK inhibitors are classified as nonselective (e.g., tofacitinib, baricitinib, peficitinib) or selective (e.g., upadacitinib, filgotinib) [7]. Each JAK inhibitor shows different selectivity for JAK isoforms and exhibits distinct pharmacological characteristics [7]. Upadacitinib and filgotinib selectively inhibit JAK1.

Increasing evidence suggests that type I interferon plays a key role in the pathogenesis of MDA5-DM [8]. JAK inhibitors block multiple cytokine signaling pathways, including interferon signaling; therefore, JAK inhibition represents a theoretical therapeutic approach for MDA5-DM. In particular, several studies have demonstrated the superior efficacy of tofacitinib for MDA5-DM compared with conventional treatments or calcineurin inhibitors [9-11]. However, mortality still exceeds 30% even with JAK inhibitor therapy.

Most studies of MDA5-DM have used tofacitinib (a nonselective inhibitor) and, rarely, baricitinib [12]. In a previous study, upadacitinib (a JAK1-selective inhibitor) was administered to a few MDA5-DM cases; however, no patients had RP-ILD at the initiation of upadacitinib therapy [13]. Here, we report a case of recurrent MDA5-DM successfully treated with upadacitinib despite worsening ILD during triple-combination therapy.

## Case presentation

A 54-year-old Japanese man with a six-year history of rheumatoid arthritis (RA) was admitted to our hospital with dyspnea, rash, weakness, and dysphagia. Nine months earlier, DM had been diagnosed at another hospital based on a characteristic skin rash, dyspnea, ILD, and anti-MDA5 antibody positivity. Induction therapy with prednisolone, tacrolimus, and six cycles of IVCY (500 mg) was effective and resulted in remission. During maintenance therapy with prednisolone (15 mg/day) and tacrolimus (3 mg/day), the patient voluntarily discontinued outpatient follow-up and treatment three months earlier. One month earlier, the patient’s dyspnea, dysphagia, and skin rash had gradually recurred, raising suspicion of recurrent MDA5-DM.

**Figure 1:**
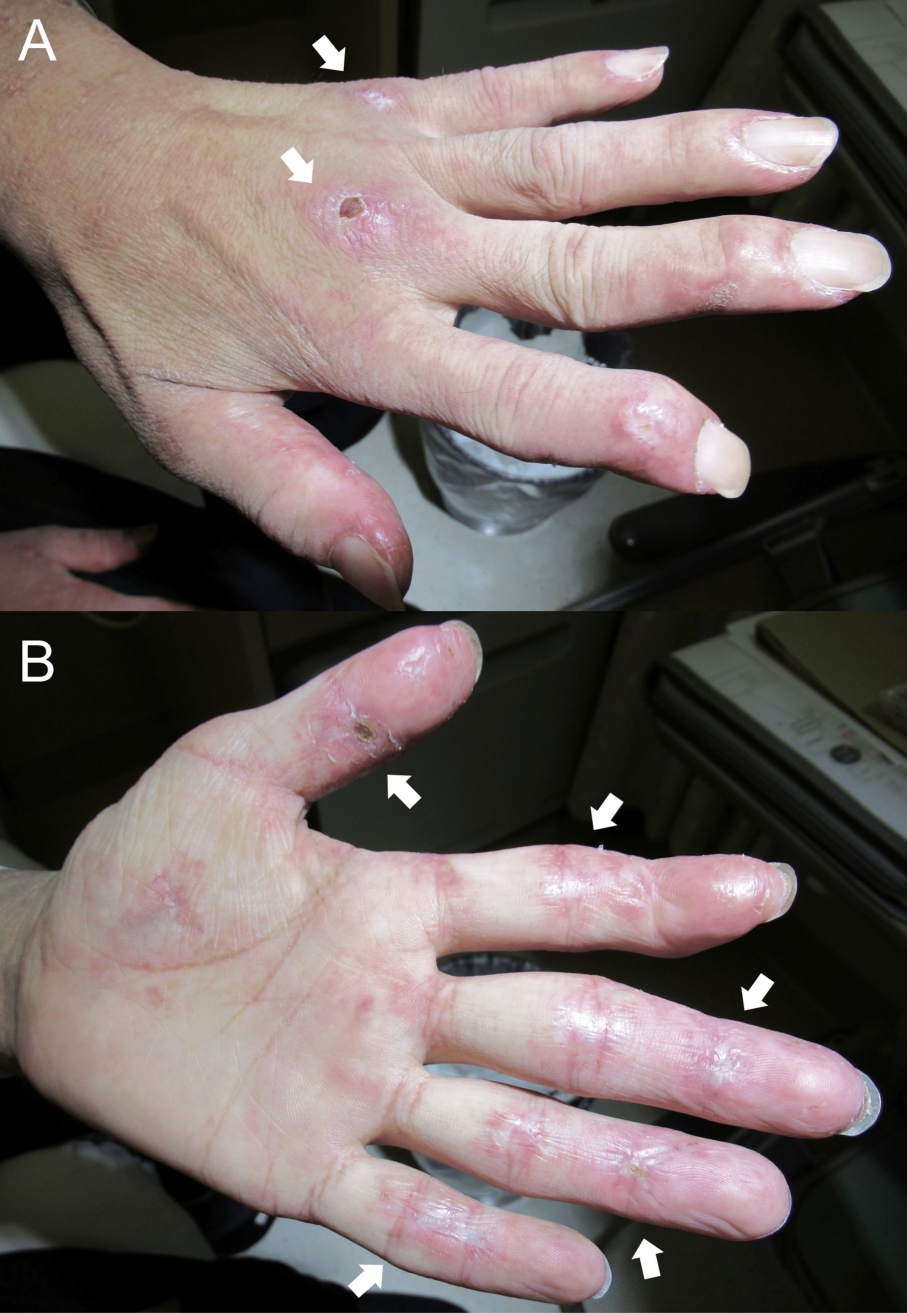
Image of the hand at admission. (A) Ulcerated Gottron’s sign was observed at the third and fifth metacarpophalangeal joints (arrows), along with periungual erythema. Swan-neck deformities were also noted. (B) Finger erythema, numerous painful interdigital ulcers, and scarring were observed on the palmar side (arrows). These cutaneous findings are typical of anti-melanoma differentiation-associated gene 5 antibody-positive dermatomyositis.

Physical examination revealed fine crackles on auscultation of both lower lung fields. Cutaneous findings included a heliotrope rash, scaly erythema over the upper arms, small punched-out ulcers on the left elbow and fingertips, Gottron’s sign on the dorsal hands, inverse Gottron’s sign on the palms, and multiple painful interphalangeal ulcers (Figure 1).

**Figure 2:**
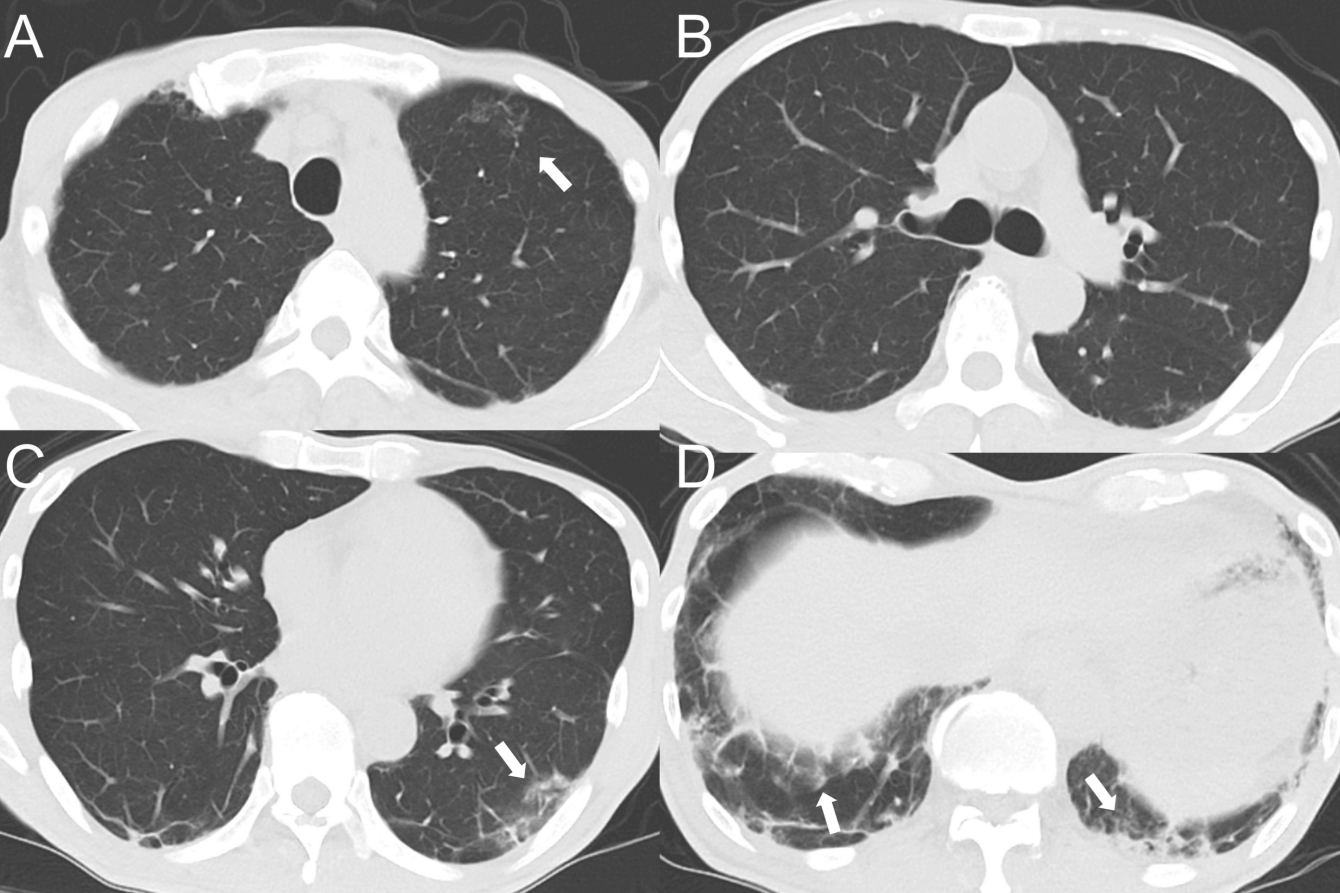
Chest computed tomography (CT) findings. Representative CT slices at four levels: (A) aortic arch, (B) carina, (C) inferior pulmonary vein, (D) lung bases. CT images show bilateral peripheral reticular opacities and ground-glass attenuation predominantly in the lower lobes (arrows).

Manual muscle testing showed mild iliopsoas weakness (grade 4/5). Laboratory findings (Table 1) showed markedly elevated serum ferritin, lactate dehydrogenase (LDH), and Krebs von den Lungen-6 (KL-6) levels of 2,090 ng/mL, 459 U/L, and 1,130 U/mL, respectively. The anti-MDA5 antibody titer was 3,900 index (normal: <32). Chest computed tomography (CT) revealed bilateral, predominantly peripheral and basal reticular opacities with areas of ground-glass attenuation (Figure 2). 

**Table 1:** Laboratory findings on admission. Abnormal values are shown in bold font. Anti-MDA5 antibody was measured by MESACUP™ anti-MDA5 antibody test (MBL, Tokyo, Japan). BUN: blood urea nitrogen, CCP: cyclic citrullinated peptide, CRP: C-reactive protein, ESR: erythrocyte sedimentation rate, IgG: immunoglobulin G, MDA5: melanoma differentiation-associated gene 5, KL-6: Krebs von den Lungen-6, RF: rheumatoid factor, SP-D: surfactant protein-D.

Blood analyses	Result	Reference range
Hemoglobin (g/dL)	10.3	12-17.6
Leukocyte (×10^9^/L)	5.13	3.9-9.8
Lymphocyte (%)	10	19-48
Platelet count (×10^9^/L)	165	130-369
ESR (mm/h)	53	0-10
Total protein (g/dL)	5.5	6.6-8.1
Albumin (g/dL)	2.4	4.1-5.1
BUN (mg/dL)	11	8-20
Creatinine (mg/dL)	0.7	0.65-1.07
Aspartate aminotransferase (U/L)	56	13-30
Alanine aminotransferase (U/L)	30	10-42
Lactate dehydrogenase (U/L)	458	124-222
Alkaline phosphatase (U/L)	73	38-113
Gamma-glutamyl transferase (U/L)	48	13-64
Creatine kinase (U/L)	135	59-248
CRP (mg/dL)	0.1	<0.14
IgG (mg/dL)	1,322	870-1,700
RF (IU/mL)	72	0-15
Antinuclear antibody (fold)	<40	<40
Anti-CCP antibody (U/mL)	196	<4.5
KL-6 (U/mL)	1,130	105-435
SP-D (ng/mL)	56.1	<110
Serum ferritin (ng/mL)	2,090	4.2-136.7
Anti-MDA5 antibody (index)	3,900	<32

No obvious honeycombing was identified. Pulmonary function testing revealed a vital capacity of 62.4%. Based on the presence of typical skin ulcers with ILD, proximal muscle weakness, elevated serum ferritin levels, and anti-MDA5 antibody positivity, the patient was diagnosed with recurrent MDA5-DM with ILD according to the European League Against Rheumatism/American College of Rheumatology classification criteria [14]. Although the patient had a history of RA, the rapid clinical deterioration, marked hyperferritinemia, characteristic cutaneous findings, and strongly positive anti-MDA5 antibody favored a diagnosis of MDA5-DM-associated ILD rather than RA-associated ILD.

**Figure 3:**
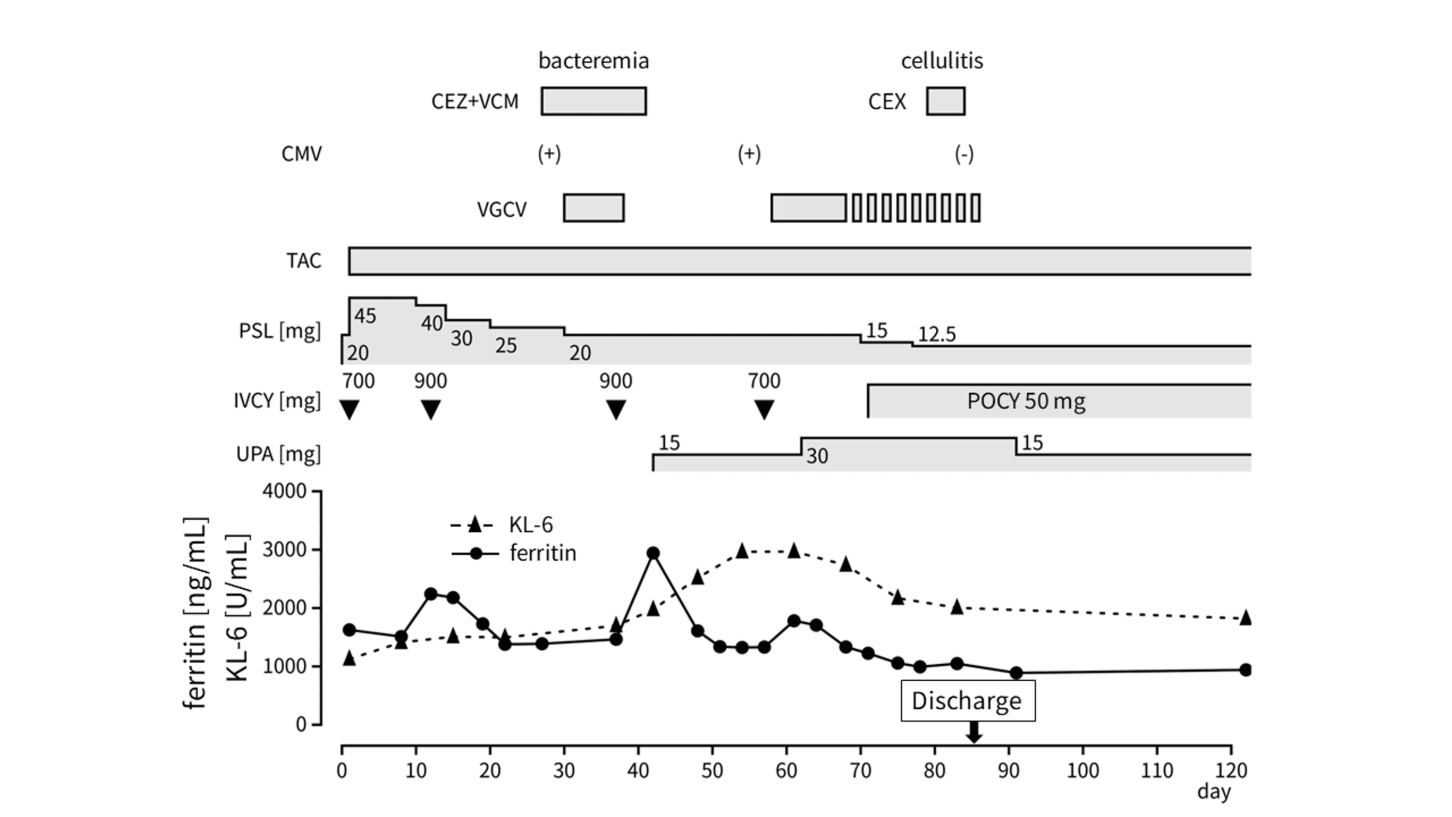
Patient’s clinical course. CEX: cefalexin, CEZ: cefazolin, CMV: cytomegalovirus antigen test, POCY: oral cyclophosphamide, KL-6: Krebs von den Lungen-6, PSL: prednisolone, TAC: tacrolimus, IVCY: intravenous cyclophosphamide, UPA: upadacitinib, VCM: vancomycin, VGCV: valganciclovir.

The patient was treated with triple-combination therapy comprising prednisolone (45 mg/day; 1 mg/kg), tacrolimus targeting a trough level >10 ng/mL, and IVCY every two weeks (Figure 3). 

**Figure 4:**
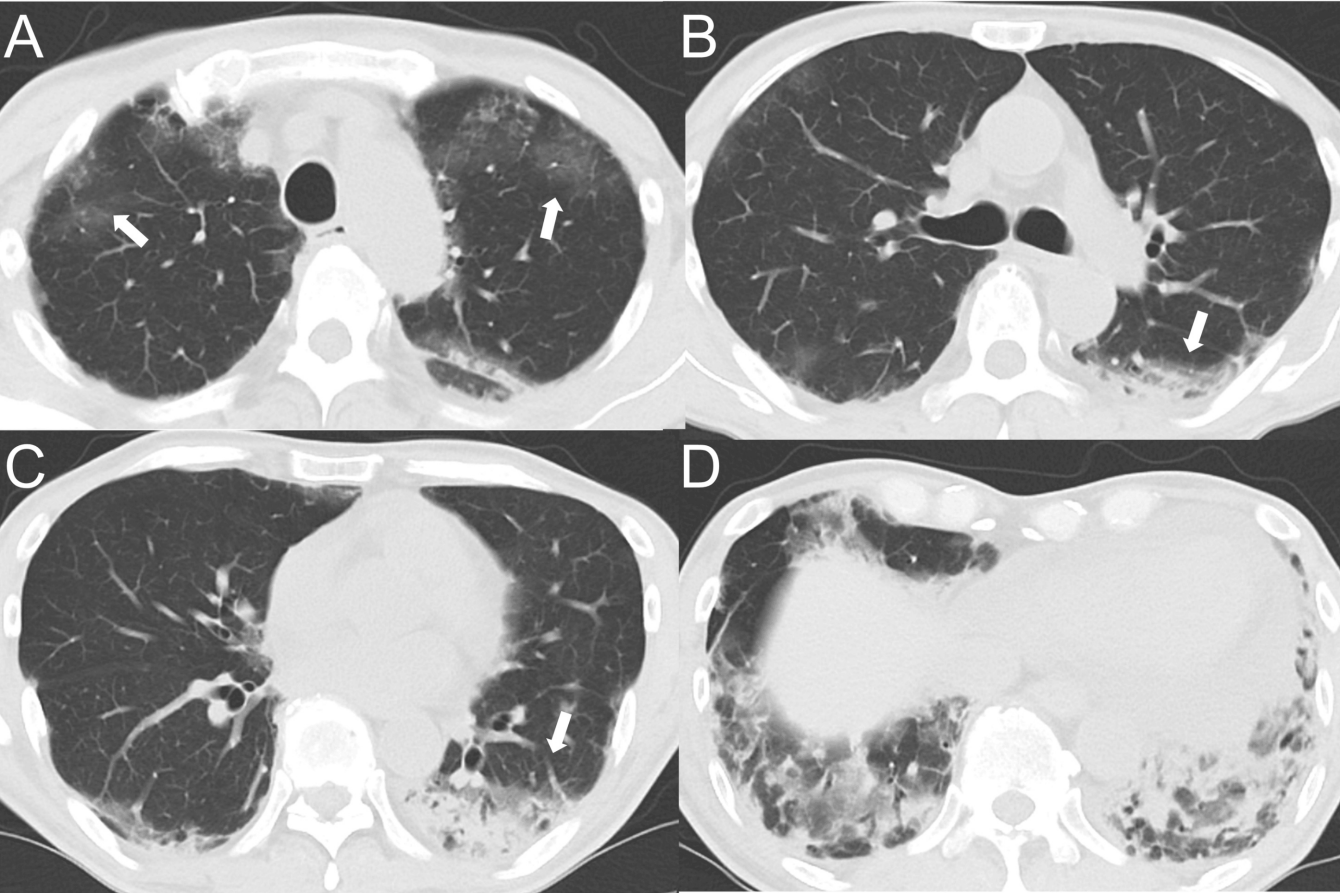
Chest computed tomography (CT) findings prior to upadacitinib administration. Chest CT images just before upadacitinib administration show newly developed ground-glass opacities in the bilateral upper lobes and worsening dense infiltrates in the bilateral lower lobes (arrows).

Although oxygen saturation was maintained at admission, the patient soon required supplemental oxygen during exertion. Given the increase in serum ferritin and KL-6 levels (2,241 ng/mL and 1,441 U/mL, respectively), the IVCY dose was increased to 900 mg. One month later, the third IVCY cycle was interrupted because of bacteremia caused by *Staphylococcus epidermidis*, necessitating intravenous cefazolin plus vancomycin for two weeks. Despite ongoing triple-combination therapy, dry cough and dyspnea gradually worsened. The patient required continuous oxygen therapy (1 L/min) and additional oxygen (2-5 L/min) during exertion. After the third IVCY cycle, chest CT revealed newly developed ground-glass opacities in the bilateral upper lobes and worsening dense infiltrates in both lower lobes (Figure 4), indicating progressive ILD. Additionally, the serum ferritin level increased to 2,942 ng/mL. Cytomegalovirus (CMV) reactivation was also observed. 

**Figure 5:**
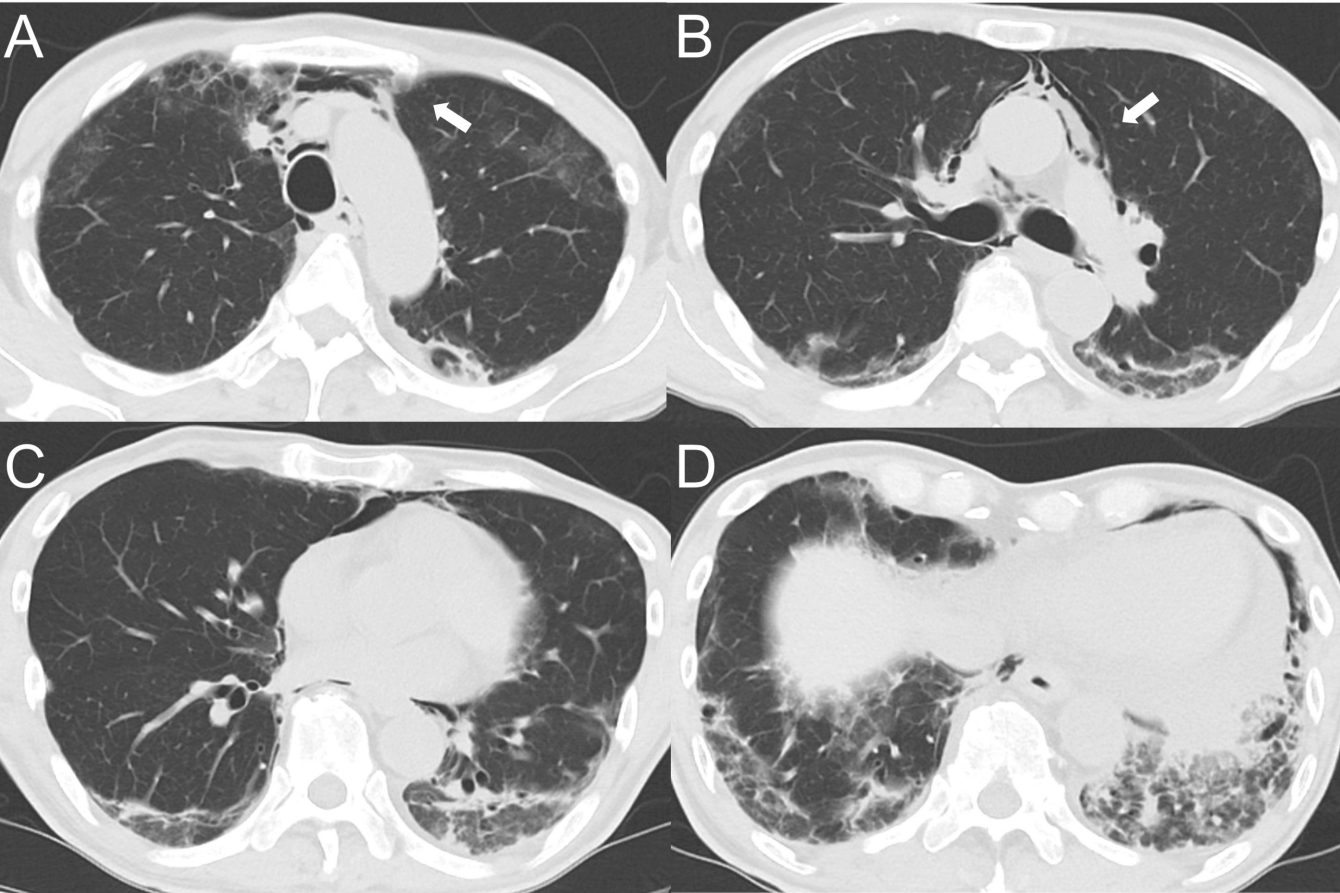
Chest computed tomography (CT) findings before discharge. Chest CT images shortly before discharge show decreased infiltrates in the bilateral lower lobes. However, pneumomediastinum developed in the upper mediastinum (arrows).

Because of worsening respiratory symptoms, deteriorating CT findings, and an increased serum ferritin level, upadacitinib (15 mg/day) was initiated. Subsequently, the patient’s persistent cough and dyspnea gradually improved, and the serum ferritin level steadily decreased to 1,330 ng/mL. CMV antigen test results were repeatedly positive, necessitating intermittent valganciclovir treatment. During upadacitinib (15 mg/day) treatment, the serum ferritin level increased again to 1,780 ng/mL. Although respiratory symptoms did not worsen, the upadacitinib dose was increased to 30 mg/day with prophylactic valganciclovir. Thereafter, serum ferritin and KL-6 levels decreased to 1,057 ng/mL and 2,170 U/mL, respectively, and continuous oxygen therapy was discontinued. The skin ulcers gradually healed, and the cough and dyspnea nearly resolved. Repeat chest CT before discharge revealed resolution of pulmonary consolidations but also mild pneumomediastinum (Figure 5).

Oral cyclophosphamide (50 mg/day) was administered instead of IVCY. Shortly thereafter, cellulitis developed around the right-hand cutaneous ulcer, which resolved after one week of oral cefalexin. The patient was discharged three months after hospitalization, with oxygen therapy required only during exertion. One month after discharge, chest radiography showed resolution of pneumomediastinum. The patient discontinued outpatient follow-up because of relocation.

## Discussion

In this case report, we describe a patient with recurrent MDA5-DM who was resistant to triple-combination therapy with high-dose glucocorticoids, tacrolimus, and IVCY. After initiation of upadacitinib, respiratory symptoms and skin ulcers gradually improved, and continuous oxygen therapy was discontinued. Increasing the upadacitinib dose to 30 mg/day further reduced the serum ferritin level. Several bacterial and viral infections developed within three months of admission and were managed conservatively.

Upadacitinib was effective in this patient who was resistant to triple-combination therapy. Serum ferritin levels increased in parallel with clinical and radiological deterioration despite triple-combination therapy. After initiation of upadacitinib, ferritin levels gradually decreased, accompanied by improvement in respiratory symptoms and radiological findings, suggesting that JAK1 inhibition may have contributed to controlling systemic inflammation and ILD progression. Poor prognostic factors for MDA5-DM include male sex, older age, hypoxia, lymphopenia, elevated LDH, C-reactive protein, CK, and serum ferritin levels, and radiographic worsening during therapy [15]. The patient exhibited several of these factors. Pneumomediastinum is also a feature of MDA5-DM and is associated with a poor prognosis [16]. Approximately half of patients who develop pneumomediastinum die within one year. The mechanism is thought to involve alveolar vasculitis or lung vulnerability due to ILD. In the present case, pneumomediastinum was mild and detected incidentally on follow-up CT. No specific intervention was required, and the patient remained clinically stable.

We chose upadacitinib because the patient had reduced renal function, likely related to the adverse effects of tacrolimus and antibiotics. Upadacitinib can be used in patients with renal impairment, which represents a notable advantage [7]. Increasing the upadacitinib dose to 30 mg/day may enhance efficacy. This concept is based on previous studies showing that increasing the tofacitinib dose from 10 mg/day to 20 mg/day may benefit some patients with MDA5-DM [17,18]. However, only a few case reports are available, and it remains unclear whether dose escalation improves efficacy without increasing the risk of adverse events.

Infections are the primary concern during JAK inhibitor treatment with triple-combination therapy. Very high frequencies of bacterial, fungal, and viral infections have been reported in patients receiving triple-combination therapy [3]. Furthermore, patients treated with tofacitinib more frequently develop viral and fungal infections, particularly CMV and varicella-zoster virus infections [5,10,11,18]. When JAK inhibitors are used to treat MDA5-DM, prophylactic valganciclovir treatment may be required [19]. Skin and soft tissue infections may increase with JAK inhibitor treatment [10]. Skin infections that could lead to bacteremia, as observed in this patient, should also be monitored because recalcitrant cutaneous ulcers can serve as entry sites [20].

Which JAK inhibitor is most effective for the treatment of MDA5-DM remains uncertain. Tofacitinib has been mostly reported in patients with MDA5-DM because it was approved earlier for treating various autoimmune diseases. Since the type I interferon family uses JAK1/TYK2 heterodimers and the type II interferon family uses JAK1/JAK2 heterodimers for signal transduction, blocking these pathways may represent an appropriate treatment strategy for MDA5-DM [7,21]. Upadacitinib strongly inhibits JAK1/TYK2- and JAK1/JAK2-mediated signaling compared with other JAK inhibitors [22]. Therefore, upadacitinib may be more effective than tofacitinib in treating MDA5-DM.

This case report has several limitations. First, the follow-up period was short because the patient relocated and discontinued outpatient follow-up after discharge. As a result, long-term outcomes and the durability of the treatment response could not be assessed. Second, detailed pulmonary function parameters and results of the 6-min walk test were unavailable during the acute phase because of the patient’s clinical condition. Repeat pulmonary function testing was not performed because of pneumomediastinum. Large-scale studies should be conducted to determine which JAK inhibitors are the most effective in patients with MDA5-DM.

## Conclusions

We report a case of recurrent MDA5-DM with RP-ILD. Upadacitinib appeared effective for RP-ILD despite an inadequate response to triple-combination therapy in this high-risk patient. JAK inhibitors, including upadacitinib, may represent a promising treatment option for MDA5-DM. This case highlights the potential role of JAK1-selective inhibition as a rescue strategy for refractory disease, even in the setting of ongoing immunosuppression. Careful monitoring and management of infectious complications are essential when intensifying immunomodulatory therapy. Further accumulation of real-world data will be crucial to better define patient selection and the risk-benefit balance in clinical practice.
